# State of quality in the COVID era including ongoing initiatives and priorities for improving quality in the future

**DOI:** 10.1093/ijcoms/lyab013

**Published:** 2021-07-29

**Authors:** Andrew Likaka, Tedla Damte, Jones Albuquerque

**Affiliations:** Ministry of Health, Lilongwe, Malawi; Keizo-Immunopathology Laboratory, Universidade Federal de Pernambuco, Pernambuco, Brazil; Keizo-Immunopathology Laboratory, Universidade Federal de Pernambuco, Pernambuco, Brazil; UNICEF Malawi, Lilongwe, Malawi; Keizo-Immunopathology Laboratory, Universidade Federal de Pernambuco, Pernambuco, Brazil

**Keywords:** covid-19, quality, equity

The African Region reported the first COVID-19 case on 14 February 2020 in Egypt and, to date, over 4.5 million cases and 120 000 deaths have been documented in all 57 countries in the continent [1]. COVID-19 has exposed huge quality gaps in the health systems in both high and low- and middle-income countries (LMICs). Patient satisfaction with care for normal medical conditions has gone down as a result of long waiting times, limitations in physician to patient contacts, disruption of services or cancellation of services due to newly introduced shift schedules, inadequate personal protective equipment, and indeed, lack of companion in COVID-19 treatment centers [2].

In Malawian context and many African countries, focus and resources have shifted from major health areas such as maternal and new born health (MNH) to COVID-19. There has been huge policy shift and donors cutting funding for MNH to support COVID-19 [3]. Even the emerging non-communicable diseases (NCDs) in Africa have received limited attention, although they contributed highly to COVID-19 mortality and morbidity. Although data shows no direct predisposition of pregnancy to COVID-19, evidence shows high likelihood of more adverse outcomes in pregnant mothers with symptomatic COVID-19 than asymptomatic cases [4]. Disruption of routine MNH services has negatively affected access to breastfeeding programs, antenatal care visits, family planning services, screening programs for reproductive cancers, such as cervical and breast cancer, and child immunization programs. Indirectly, COVID-19 has contributed to poor outcomes in the delivery of quality maternal and new born as well as child health services. It will be imperative to explore the impact of COVID-19 on institutional delivery, delivery by skilled personnel, comparative analysis of home deliveries and hospital deliveries, the perception of pregnant women on the quality of care provided during COVID-19, and the estimations of maternal and neonatal deaths directly and indirectly associated with COVID-19.

COVID-19 has negatively impacted on the quality of health services provided by health care workers as it contributed to shortages in various health facilities [5]. Prevalence of COVID-19 amongst health workers has been reportedly high, up to 10% in Qatar, for example [6], and over 150 000 infections and 1400 deaths were reported during the early days of the COVID-19 pandemic among the health care workers [5]. The situation has been worse in LMICs where human resources for health shortage was already a challenge for the provision of quality of care. With various changes in infection prevention and control guidelines and protocols for health care workers, health facilities have experienced closures due to infected care providers who had to go into isolation for 14 days with all their contacts [7]. Although it has been difficult to detect the source of the infections amongst health workers, community transmission is likely more of a cause than transmission within health facilities. Most chronic conditions and elective surgeries were postponed, thereby causing more harm in patients and leading to dissatisfaction [2]. The use of students to cover for the shortages in many countries need to be evaluated to see whether it impacted negatively or positively on patients’ outcomes. It will be necessary to look at the roles of patient guardians in COVID-19 treatment centers as it can ease on the pressure of work among health workers. Another area to be explored is the impact of incentives to health workers in providing quality of care during COVID-19. Many countries have seen innovation and technology being utilized to provide support during health worker shortages as well as help reduce risk for infections among health care workers. Rwanda, for example, introduced the use of robots in the COVID-19 treatment centers as a way of minimizing the risk of contact between staff and patients [8]. It will be imperative to look at the cost-effectiveness and scalability of such innovations in Africa. Furthermore, COVID-19 has exposed how vulnerable health care workers are; thus, defining the minimum standards for health worker safety and best methods for enforcement is pertinent.

Digital health has proved to be an opportunity during the COVID-19 pandemic to improve the quality of care by improving access to health services, providing real-time data for decision-making and, indeed, providing information to rural populations within a short period. However, there is still more room for improvement in redesigning digital health systems toward patient centeredness, user centeredness, and, finally, data centeredness in that order. COVID-19 has seen the deployment of several digital health applications to LMICs but they are now faced with the challenge of sustainability. It is encouraging that the need to use data for decision-making among the public has grown with most of these applications. Moving forward, we must evaluate the cost of deploying digital health systems during a pandemic, analyze challenges with the deployment of digital health platforms in LMICs, especially during crises, and document opportunities brought in by digital health.

Quality, equity, and dignity are key words when it comes to analyze whether the health system has been responsive to the needs of the public. During COVID-19, the world faced huge challenges with the availability of personal protective equipment (PPE), with priority being given to developed and high-burden countries. Access to the COVID-19 vaccines in LMICs has been a huge challenge, clearly exposing the gaps in equitable access to life-saving interventions. COVID-19 has highlighted the inequities existing in the delivery of health services; the rich still have higher access to quality health services than the poor [9]. With the global call for Universal Health Coverage, equity is paramount. No one should be left behind due to their socioeconomic status.
